# Helping to improve the practice of cheminformatics

**DOI:** 10.1186/s13321-017-0217-z

**Published:** 2017-06-14

**Authors:** Rajarshi Guha, Egon Willighagen

**Affiliations:** 1 National Center for Advancing Translational Sciences, 9800 Medical Center Drive, Rockville, MD 20850 USA; 2 Department of Bioinformatics - BiGCaT, NUTRIM, Maastricht University, P.O. Box 616, UNS 50 Box 19, 6200 MD Maastricht, The Netherlands

As we take over the editorial reigns of *J. Cheminform.* we would like to present our goals for the journal and our views on where cheminformatics as a field is headed and how the journal may influence it. As editors our overarching goal is to disseminate cheminformatics research and practice that is impactful and useful within the community. Open Access publishing remains central to this, as well as continued innovation in simplifying reuse of published work. This includes encouraging FAIR [1] outputs and clear, open licensing, resulting in research products that can be reused and reproduced. Recent updates to the journal’s website exemplify our attempts to encourage reuse of data from articles (e.g. integration with FigShare).

In parallel with good publishing practice, we want to focus the journal on innovative research in, and the practice of, cheminformatics. This leads to an obvious question: what do we consider cheminformatics? An answer to this allows us to judge where innovation lies. Very broadly, cheminformatics addresses the generation, representation and manipulation of chemical information, with the chemical structure at its foundation. And thus innovation can occur at multiple levels, starting from the chemical structure itself (non-graph representations, learned representations) to novel ways to integrate chemical information with other data types (such as sequence data or protein structure data). At the same time, it is important to understand the bottlenecks and challenges in dealing with chemical information. While not necessarily novel, discussion of how organizations deal with chemical information problems and practice cheminformatics, which tools work and which do not, are all valuable contributions to the field.

## Cheminformatics practice

Given the broad definition, it is useful to focus on a few areas that we feel are relevant to the future of the field. This aspect was touched upon by Wild [2] and more recently by others [3, 4], who focused on specific challenges, many of which remain open. Taking a broad view, we believe that the future of cheminformatics lies in addressing large scale chemical information, across different domains. Particularly, the increased size of chemical space that can be explored, either explicitly [5] or implicitly [6] leads to opportunities in many areas of cheminformatics including property prediction, searching and representing such massive chemical spaces. Given the interdisciplinary nature of the field [7], many of the problems associated with this vision will require a combination of expertise in computer science, statistics, chemistry and biology and we hope to encourage scientists, who are not necessarily chemists, to contribute their expertise via collaborations. While a vision provides us direction, it is useful to point out specific areas that we are actively looking to publish in. Examples include:

- New methodology and work that address theoretical underpinnings of cheminformatics methods, such as new representations to complement QM and chemical graphs and methods to derive them;
- Approaches to describe and model new data types such as compound combination responses;
- New topical areas, such as approaches to modeling and screening inorganic materials or informatics approaches to QM/MM problems that enables application to large datasets; and
- New perspectives on how chemical information can and should be employed in multi-disciplinary settings such as drug discovery and development pipelines, clinical environments or regulatory settings.

Importantly, this partial list is driven by our scientific interests and vision. It is not comprehensive and we encourage authors to contact us regarding topics not listed here.

## Publishing practice

While aiming for high quality, relevant and innovative topical content is a primary goal, it is equally important that we ensure the usability and accessibility of the content published by the journal. Our goal is to publish material that can be reused, modified, and redistributed as far as possible. For studies that involve proprietary datasets, we encourage authors to include at least one non-proprietary dataset so that users can reproduce the study. Similar requirements for software usage are described elsewhere (https://jcheminf.springeropen.com/submission-guidelines/preparing-your-manuscript/software).

We aim to follow, and encourage authors to follow, FAIR principles, which outline how research output is findable, accessible, interoperable, and reuseable (e.g. reflected by the participation in the Initiative for Open Citations). As an Open Access journal, open licensing of content (including data and code) is a foundational aspect and coupled with waivers, we hope to further remove barriers to adoption of material published in the journal and encourage authors from around the world to consider *J. Cheminform.* as a venue for their work.

At the journal level, we will continue to work on improving dissemination of research published in *J. Cheminform.* using a variety of mechanisms such as videos, blog posts and Twitter (https://twitter.com/jcheminf). We are actively reviewing article and reviewer guidelines to ensure clarity in terms of what is or isn’t acceptable for different article types. More generally, as a platform for the dissemination of computational research we hope to contribute to the solution of challenges described by Neylon et al. [8] and List et al. [9]

## Conclusion

While pontificating on the future is always a fun task, we are committed to the more challenging task of bringing the future to the present. To that end, we are currently designing a series of thematic issues and actively soliciting articles in a number of the areas listed above. In these efforts, the Editorial Board has provided useful inputs and we would like to acknowledge their participation in the journal’s activities. Simultaneously, we are working with the publisher to make our content more accessible in more ways.

We believe that cheminformatics has a bright and broad future and that by focusing on emerging topics, cheminformatics practice and ensuring Open publishing policies, *J. Cheminform.* will play a key role in pushing the field forward.
